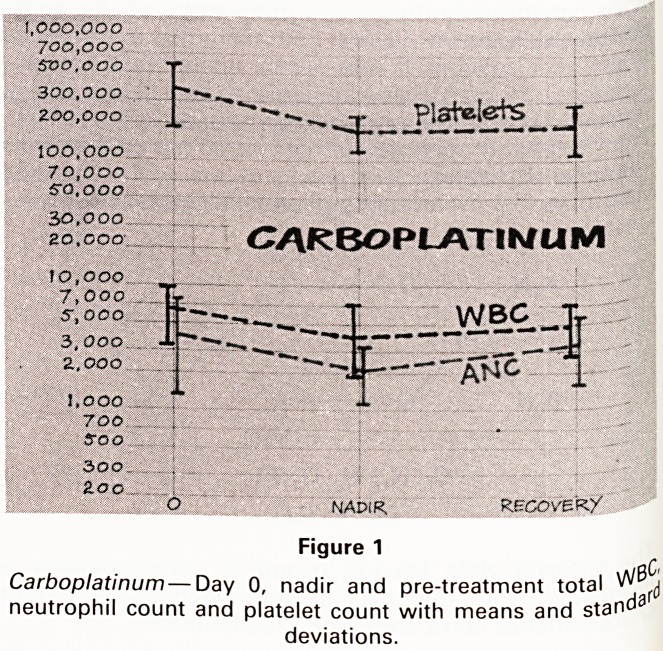# Carboplatinum

**Published:** 1988

**Authors:** J. Chambers, A. Oakhill, M. G. Mott

**Affiliations:** Bristol Children's Hospital; Bristol Children's Hospital; Bristol Children's Hospital


### Carboplatinum in Childhood Cancer

J. Chambers, A. Oakhill, M. G. Mott.
Bristol Children's Hospital

### INTRODUCTION

Carboplatinum is one of a series of Platinum compounds synthesised with the aim of finding a Cisplatinum analogue with equivalent or superior anti-tumour activity but reduced renal toxicity and less emetic activity.

Cisplatinum was first used in clinical studies in the early 1970's and is established as essential in the treatment of malignant germ cell tumours particularly teratomas. It is, however, associated with severe toxicity: in particular renal, (raised creatinine in 20% patients); peripheral neuropathy in >20%; ototoxicity in 20%, and severe nausea and vomiting which are difficult to control even with high dose antiemetic combinations. Ever since its clinical usefulness was confirmed the search has been on for a platinum compound with equivalent therapeutic efficacy but less toxicity.

### MECHANISM OF ACTION OF CISPLATINUM

Cisplatinum is thought to act by inhibiting DNA synthesis. It is activated by hydrolysis with dissociation of the leaving chloride group. The reaction with DNA results in interstrand and intrastrand cross-linking and DNA protein cross-linkings. The cis transfiguration of the molecule is necessary to form a stable compound with DNA.

CISPLATIN

This is the formula for Cisplatinum and the chloride groups are referred to as the leaving groups.

It is the characteristics of the leaving groups which are thought to be critical both for toxicity and activity. Many platinum compounds have been studied and discarded but two compounds known as JM8 and JM9 were selected for further study on the basis of their activity against transplantable rodent tumurs. JM8 was also active against human lung xenografts and was chosen for clinical evaluation.

### CARBOPLATINUM (JM8 Paraplatin)

**Chemical Structure:**

diammine (1,1-cyclobutane dicarboxylate) platinum

$C_6H_{12}N_2O_4Pt$

PARAPLATIN

The dicarboxylate leaving group in carboplatinum is more stable than that in cis platinum leading to different characteristics compared with Cis platinum.

**Pharmacokinetics**
The pharmacokinetics of carboplatinum differ significantly from cis platinum.

a) It is more stable in human plasma leading to less irreversible protein binding.
b) More carboplatinum is excreted by glomerular filtration – a greater percentage of a given dose is present in the urine and hence a smaller percentage of total platinum remains in the body.
c) The terminal half life of free non-protein bound platinum is approximately ten times that of cis platinum.
d) The terminal half life of total platinum is measurable in hours and is approximately fifteen times shorter than that of cisplatinum.

**Excretion**
The major route of excretion is via the kidney and total body and renal clearances of free platinum correlate with the Glomerular Filtration Rate (GFR). In man, as observed in animals, and in contrast to cis platinum, there does not appear to be any tubular secretion. Phase I studies have indicated that toxicities correlate well with creatinine clearance and that the percentage reduction in platelet count correlates highly and linearly with the area under the curve of plasma ultrafilterable platinum. A more predictable myelosuppression is obtained by correlating the dose with GFR.

**Pre-clinical studies**
Pre-clinical studies of carboplatinum showed activity comparable to cis-platinum in many tumour lines, superior in some and less in a few.

**Clinical Studies**
Phase I and II studies were done at the Royal Marsden Hospital from 1981. 69 patients were entered into a Phase I study[1], 16 of whom had renal impairment. There was no evidence of nephrotoxicity or ototoxicity, nausea and vomiting were less than with cis platinum, and the dose limiting factor was myelosuppression.

Phase III studies in adults[2]: carboplatinum has been compared with cisplatinum in a randomised study of women with ovarian carcinoma. Its reduced toxicity and equivalent therapeutic benefit was confirmed.

Dosage escalation studies in patients with stage IV ovarian carcinoma have also been done[3]. The principal toxicities were bone marrow suppression, particularly thrombocytopenia and nausea and vomiting. No neurotoxicity was seen and alopecia was rare.

**Bristol Children's Hospital Study**
From April 1986, selected patients with poor prognosis tumours or who had relapsed, were entered into the Bristol Children's Hospital Resistant Tumour Protocol (BCH RTP) below.

### BCH RESISTANT TUMOUR-PROTOCOL I

| | | |
|---|---|---|
| 1. | *Vincristine* | 1.5 mgs/$m^2$ (max 2 mg) weekly × 7 doses during the first cycle, than every 3 weeks. |

**Table 1**

| *Diagnoses* | *No. of Pts.* | *1° Treatment* | *Relapse* | *Previous Chemotherapy* |
|---|---|---|---|---|
| Ewing's sarcoma | 4 | 3 | 1 | 1 |
| Neuroblastoma | 3* | 2 | 1 | 1 |
| Osteosarcoma | 3 | 3** | — | 1 |
| Rhabdomyosarcoma | 2 | 2 | — | — |
| Wilm's | 1 | — | 1 | 1 |
| Sacroccygeal teratoma | 1 | — | 1 | 1 |

*One inoperable ganglioneuroma.
**Had chemotherapy schedule changed because of non-response at time of biopsy.

2. *Ifosfmaide* 6 G/m$^2$ as a 24 hour infusion.
   *MESNA* 6 G/m$^2$ as a 24 hour infusion.
   *VP 16* 150 mgs/m$^2$ daily × 3.
3. *Epirubicin* 150mgs/m$^2$ i.v. over 15–30 minutes.
4. *Carboplatin* 500 mgs/m$^2$ i.v. over 15–30 minutes.

2,3, and 4 given at three week intervals (9 week cycle)×5.

Carboplatinum is the third arm of this protocol and is given at a dose of 500 mg/m$^2$ in combination with vincristine 1.5 mg/m$^2$, 3 weeks after Epirubicin.

Between September 1985 and January 31st 1987, 14 patients aged between 4 and 17 have been treated with 39 courses of carboplatinum. In 12, the carboplatinum was given as part of the BCH RTP above. The first 2 patients received carboplatinum at doses of 300 mg/m$^2$ and 400 mg/m$^2$ respectively prior to the commencement of the protocol.

Five of the 14 had received prior chemotherapy. The diagnoses and characteristics of the patients are listed in Table 1.

**Method of Administration**

After full blood count and creatinine measurements, the vincristine is given as an i.v. push and carboplatinum is then added to 100 ml of 5% dextrose and given over 1 hour. It is given on an outpatient basis with an i.v. anti-emetic given at the end of the infusion and a supply of anti-emetics (usually suppositories) given to the patients for subsequent use as required. Most patients go home the same day, though a few elect to remain in hospital overnight because of vomiting.

Full blood counts are done at approximately 10 days and biochemical investigations, including creatinine, LFTs calcium and magnesium prior to the next course of chemotherapy. Audiograms were carried out where possible before each course.

**Toxicity**

a) *Haematological*

There was remarkably little haematological toxicity with nadir neutrophil counts falling below 1×10$^9$/L on only 4 occasions and the nadir platelet count below 100×10$^9$L on 7 occasions. Table 2 shows the nadir values for total white count, neutrophils and platelets.

**Table 2**

**Nadir values following carboplatinum**

| | *Range* | *Mean* | *Median* |
|---|---|---|---|
| Total WBC×10$^9$/L | 0.8–10.1 | 3.9 | 3.3 |
| Neutrophils×10$^9$/L | 0.1–5.4 | 2.13 | 1.95 |
| Platelets×10$^9$/L | 40–366 | 173 | 156 |

Table 3 shows the time in days for nadir to be reached. It was late for the platelet count, more than half the patients actually experiencing their nadir platelet count at the time of their next chemotherapy. This led to treatment delays in 5 out of 39 courses—2 for neutropenia and 3 for thrombocytopenia.

**Table 3**

**Time in days to nadir from carboplatinum treatment**

| | *Range* | *Mean* | *Median* |
|---|---|---|---|
| Total WBC | 8–22 | 12 | 11 |
| Neutrophils | 8–21 | 13 | 10 |
| Platelets | 8–21 | 17 | 21 |

Figure 1 shows graphically the Day 0, nadir and next pre-treatment white count, neutrophil and platelet counts with the means and standard deviations.

There was only one documented case of fever and neutropenia in this group of patients following carboplatinum. Eight of the fourteen patients had long lines in situ.

b) *Vomiting*

All patients vomited despite receiving prophylactic antiemetics. The most common pattern was for moderate-severe vomiting to start approximately 5–6 hours after the carboplatinum and to continue for

**Figure 1**

*Carboplatinum*—Day 0, nadir and pre-treatment total WBC, neutrophil count and platelet count with means and standard deviations.

2–10 hours at home. Appetite returned to normal after approximately 36–48 hours.

c) *Renal*

No child who had not previously received cis platinum had evidence of renal toxicity as measured by serial serum creatinine levels. One child, previously treated with cis platinum to a total dose of 420 mg 3 years prior to carboplatinum, had grade I (WHO) elevation of serum creatinine at the completion of 4 cycles of carboplatinum at 300 mg/m$^2$ in December 1985. By January 1987 $^{51}$CrEDTA clearance showed his GFR to be marginally reduced. Serum magnesium has been measured after 17 courses and there has been no reduction.

d) *Audiological*

There was no demonstratable change in audiograms done on patients on the protocol. The child who had had previous cis platinum therapy had bilateral high frequency loss which has remained stable since carboplatinum therapy was completed 15 months ago.

e) *Liver*

There was no evidence of liver toxicity as measured by changes in liver function tests such as bilirubin and aspartate transaminase.

### CONCLUSIONS

Carboplatinum can be given to children at a dose of 500 mg/m$^2$, on an out-patient basis, is reasonably well tolerated (vomiting can usually be managed by parents at home) and does not cause unacceptable haematological toxicity. There was no evidence in these patients of renal, hepatic or ototoxicity. Alopecia was already present in all patients and so it is not possible for us to evaluate this though it was reported to be rare in one adult study.

### REFERENCES

1. A. H. CALVERT, S. J. HARLAND, D. R. NEWELL, Z H SIDDIK, K R HARRAP. (1985). Phase I studies with carboplatinum at the Royal Marsden Hospital. *Cancer Treatment Reviews* **12**, 51–57.
2. E. WILTSHAW. (1985). Ovarian trial at the Royal Marsden. *Cancer Treatment Reviews* **12**, 67–71.
3. E. WILTSHAW. Conventional and high dose studies in ovarian cancer. *Symposium on Paraplat.* April 7th, 1986.

## Figures and Tables

**Figure 1 f1:**